# A Review of Dementia Patients in a Local Neuro‐cognitive Clinic

**DOI:** 10.1002/alz.088928

**Published:** 2025-01-09

**Authors:** King Son LAI

**Affiliations:** ^1^ Hospital Authority, HKSAR, nil Hong Kong

## Abstract

**Background:**

It is more ideal to manage dementia patients in memory clinics but not all of them are referred to such clinics. Also, the characteristics of these patients are less studied and local published data is limited. In 2004, the Neuro‐cognitive Clinic of Alice Ho Miu Ling Nethersole Hospital was established. Situated inside the Geriatric Day Hospital, this clinic receives full support from other allied health departments (e.g. physiotherapy and occupational therapy departments). It serves patients living in the district presenting with neuro‐cognitive problems, who are attended by geriatric specialists or higher trainees in geriatric medicine under specialists’ supervision.

**Objective and Method:**

It is a retrospective study to analyse the characteristics of patients attending this clinic in the past 10 years and their changes in function over time. The demographic data, medical comorbidities, mobility status, functional level, neuro‐cognitive and neuro‐psychiatric functions of the patients at our clinic were reviewed and the changes over three years were analysed.

**Results:**

Among the 320 patients of age 65 or above, over 60% were female. More than 90% of them presented with memory deficit as the chief complaint. Alzheimer’s disease (AD) was the commonest dementia subtype, representing nearly half of all the cases, and was followed by mixed dementia and vascular dementia. The mean MMSE score at initial presentation was 16.4. Hypertension, hyperlipidaemia, diabetes mellitus and history of cerebrovascular diseases (stroke) were common medical comorbidities. Nearly all patients had an initial radiological neuroimaging for evaluation, mostly computed tomography scan of the brain. While findings of small vessel disease, lacunar infarcts and periventricular ischaemia largely predominated in vascular dementia patients (83.0%), such findings were also found substantially in AD patients (50.3%) in contrast to cerebral atrophy (18.3%) and normal (25.5%). More than half of all patients were prescribed with anti‐dementia drugs. AD patients taken anti‐ dementia drugs showed a slower decline of MMSE score at three years than those without anti‐dementia drugs.

**Conclusion:**

This study provided some important data on the characteristics of dementia patients in a local neuro‐cognitive clinic in various aspects, and many of them were comparable with other published international studies.

